# A Novel Strategy for Assessing Bone Marrow Plasma Cell Percentage: Development and Internal Validation of a Surrogate Calculation Approach

**DOI:** 10.1155/ah/1191575

**Published:** 2025-11-14

**Authors:** Ethan James Gantana, Zivanai Cuthbert Chapanduka

**Affiliations:** ^1^ Department of Pathology, Stellenbosch University, Cape Town, South Africa, sun.ac.za; ^2^ Department of Hematology, National Health Laboratory Service, Cape Town, South Africa, nhls.ac.za

## Abstract

**Introduction:**

The differentiation and diagnosis of plasma cell (PC) neoplasms (PCNs) such as multiple myeloma (MM) rely on the quantification of clonal PCs in the bone marrow (BM). For monitoring, the International Myeloma Working Group (IMWG) defines stringent response criteria based on the percentage of BM PC. However, BM biopsies are invasive and painful, and often with sampling variability. This study investigates whether routine biomarkers can predict BM trephine (BMT) PC% using multivariate regression.

**Methods:**

A cross‐sectional study was conducted at Tygerberg Hospital, South Africa. Data were extracted from the National Health Laboratory Service (NHLS) database. The final dataset included 112 newly diagnosed MM patients with complete biomarker data for training of the partial least squares regression (PLS‐R) model. Variables analyzed included SFLC ratio, paraprotein, Hb, calcium, creatinine, and albumin. Statistical methods included correlation analysis, regression modeling, and internal validation.

**Results:**

The cohort had a median age of 61 years and a male‐to‐female ratio of 1:1.5. PLS‐R analysis identified significant predictors of BMT PC%, including SFLC ratio, paraprotein, Hb, and serum albumin. The final model equation showed moderate predictive power (*Q* ^2^ = 0.410, *R* ^2^ *Y* = 0.432). Spearman correlation analysis showed a moderate positive relationship (*p* = 0.585) between predicted and actual BMT PC%. Linear regression analysis also confirmed that BMA PC% (*R* ^2^ = 0.489) was a stronger predictor of BMT PC% than flow cytometry PC% (*R* ^2^ = 0.184).

**Conclusion:**

This study provides proof of concept for the use of biochemical markers to predict BMT PC% and offers a less invasive alternative for PC quantification in PCN. Standardization of sampling and measurement of biomarkers is essential for the refinement of these predictive models. Future multicenter studies should include prospective data collection to improve model accuracy and clinical applicability.

## 1. Introduction

The differentiation between and diagnosis of plasma cell (PC) neoplasms (PCNs) depend on the accurate quantitation of clonal PCs in the bone marrow (BM), which is traditionally performed on BM biopsies [1, 2]. A combination of ≥ 10% clonal PCs in the BM, as well as other laboratory parameters and radiological imaging, remains the most important investigation for the initial diagnosis of multiple myeloma (MM) and other PCNs [2, 3]. Prognosis and treatment response assessment, which guide treatment protocols, are also determined by the percentage of PCs in the BM, particularly with regard to the International Myeloma Working Group (IMWG) standard criteria for complete and stringent complete response [2].

In 97% of patients with MM, the clonal PC secretes an excess of monoclonal proteins (“paraprotein”) and/or free light chains into the circulation, which can be measured using biochemical techniques [4]. The same proteins are responsible for the renal damage (light‐chain cast nephropathy) associated with PCN, which can be measured by serum creatinine and estimated glomerular filtration rate (eGFR) and is regarded as the only renal failure that should be considered a myeloma‐defining event (MDE) [5, 6]. A presumptive diagnosis of light‐chain cast nephropathy can be made on the basis of a high involved FLC level (> 1500 mg/L) [6]. Patients with smoldering MM who had an involved free light chain level of at least 1000 mg/L were found to have a risk of progression to MM of 82% at 2 years [7]. Anemia in PCN is predominantly the result of reduced erythropoiesis as a consequence of PC infiltration in the BM. Its use as a MDE should therefore be undertaken with caution, and the degree of PC infiltration should be correlated with the anemia [6]. These biomarkers are used for risk stratification and diagnosis of PCN [8, 9].

Although biomarkers are useful in monitoring patients during follow‐up, BM biopsies are still performed at certain follow‐up appointments at the discretion of the physician to look for additional causes of cytopenias and to determine whether PC tumor burden has improved in patients receiving inferior chemotherapy regimens. This is particularly true in low‐ to middle‐income countries where the standard three‐drug combination chemotherapy (with or without daratumumab) is not readily available upfront for patients who are transplant eligible [10].

BM biopsy is an invasive procedure associated with the risk of discomfort, pain, and other complications, which may limit its utility for serial monitoring and routine clinical use [11, 12]. Moreover, BM histology requires expertise and may be subject to sampling bias or variability, potentially leading to underestimation or overestimation of disease burden. This poses considerable diagnostic and therapeutic challenges. Furthermore, there is no completely satisfactory reference method for the quantitation of PC in BM. Primarily, pathologists rely on BM biopsies of adequate quality supported by immunohistochemical (IHC) stains such as CD138, kappa, and lambda. Each of these methods has its own advantages and disadvantages and varying degrees of accuracy, which have been extensively reviewed [3, 13–19]. In this context, the development of a less invasive method for estimating PC count was the rationale for this study.

Because of the effect of biomarkers on the extent of disease and vice versa, and because they are already used to assess response at earlier stages of disease, we propose to incorporate these biomarkers into mathematical models and algorithms such as a partial least squares regression (PLS‐R) model to predict BM trephine (BMT) PC percentage counts and assess disease burden at clinical follow‐up. This model could potentially utilize a combination of biomarker variables that are measured at the time of the BM biopsy, such as hemoglobin (Hb) level, serum protein electrophoresis (SPEP) values, serum‐free light chain (SFLC) ratios, and serum biochemical tests such as calcium, albumin and creatinine. All these variables are either directly or indirectly related to the diagnosis of MM [10]. The tests that determine the values of these variables are easily accessible through routine laboratory testing on peripheral blood samples, which offers the advantage of being less invasive, more cost‐effective, and convenient than BM biopsies.

This original research serves as a proof of concept for the development of a reliable and standardized approach for surrogate assessment of the percentage of PCs in the BMT (BMT PC%) in PCNs. The study investigates whether biomarker‐based calculations, including paraprotein concentration (measured by SPEP), involved‐to‐uninvolved SFLC ratio, serum creatinine, Hb, serum calcium, and albumin, can accurately predict PC percentage in BM biopsies.

## 2. Method

This is a cross‐sectional study conducted at the Division of Hematology and the National Health Laboratory Service (NHLS) at the Faculty of Medicine and Health Sciences (FMHS), Stellenbosch University, Tygerberg Hospital (TBH). TBH, 1853 bed facility, serves as the main teaching hospital for Stellenbosch University’s FMHS and derives its patient population from the Western Cape and Eastern Cape Provinces of South Africa. Patients diagnosed with MM between 1 January 2016 and 30 November 2024 were included in the study if they were ≥ 18 years of age, flow cytometry (FC) was performed on a BM aspirate (BMA) sample, and a CD138 IHC–stained BM biopsy was available. Data were collected from the NHLS laboratory information system (LIS), TrakCare by InterSystems Corporation (Cambridge, MA, USA). Data were extracted from the LIS using the PC Dyscrasia FC Panel (“PCDP/H520”) test code performed at the time of diagnosis of MM. A total of 218 patients were identified using the “PCDP” test code, and 152 patients were eligible for data analysis. A total of 66 patients were excluded from the study because, although a “PCDP” test was registered on TrakCare for them, the patients were diagnosed with a hematological malignancy other than MM, FC was performed on a sample other than the BMA, no BMT biopsy was available, or the test was performed on a follow‐up patient and not for diagnostic purposes (Figure S1). Of the 152 patients included in the study, the data from 112 patients were used in the PLS‐R training dataset, as these patients had no missing data points.

Data collected included demographics (age and sex), laboratory‐defined MDEs, immunoglobulin (Ig) isotypes, and BM PC (BMPC) percentage from reported BMT biopsies, FC results, and myelogram assessments on BMA smears. Radiological imaging or other clinical data were not collected for this study.

Statistical analysis included correlation calculations, which were used to determine the relationships between the variables and BMT PC%, the assessment of the importance of the variables in the projection (variable importance in projection [VIP] scores), and the PLS‐R on the training dataset (*n* = 112) to develop an equation model for predicting the BMT PC% using the laboratory‐based biomarkers. The quality of the model was assessed using the *Q* ^2^, *R* ^2^ *Y*, and *R* ^2^ *X* cumulative indices. During internal validation, the model predictions on the same training dataset were compared with the reported BMPC% for each sample to evaluate the correlation and accuracy of the predictions using the equation model. Normality tests were then performed on the validation dataset to determine which correlation coefficient would be best to assess the performance of the PLS‐R equation model.

A linear regression analysis was also conducted for each of the reported BMA PC% and reported FC PC% as the quantitative variable (*X*), respectively, and the reported BMT PC% as the dependent variable (*Y*) to develop an equation model to predict BMT PC% using either the reported BMA or FC PC%.

All equation models were tested using the same dataset that was used to develop the model, and correlation coefficients were calculated to assess the relationship between the predicted and reported BMT PC% values.

Statistical analysis and graphical visualization of the data were performed using XLSTAT software (Data Analysis and Statistical Solution for Microsoft Excel, Addinsoft, Paris, France, 2021) and Microsoft Excel [20].

Data management ensured patient confidentiality through anonymization, password‐protected electronic data, and secure storage. Ethical approval was granted by the Health Research Ethics Committee of Stellenbosch University (HREC reference number: N24/09/107).

## 3. Results

### 3.1. Demographics and MDEs

A total of 152 patients were included in this study, with 61 males and 91 females and a male‐to‐female ratio of 1:1.5. The mean/median age at diagnosis was 61 years (range: 31–83 years), and the peak frequency was in the 52–61 years age group, followed by the 62–71 years age group (Table 1). The most frequent MDEs (Table 1) were anemia (67%) and the presence of ≥ 60% clonal PCs in the BM (63%). Only three patients with a serum creatinine level of > 177 μmol/L (*n* = 34) had an involved serum FLC level of < 1500 mg/L. The IgG isotype was most commonly seen in 90 patients (59%), the IgA isotype in 26 patients (17%), light chain–only disease in 27 patients (18%), nonsecretory MM or no definite/quantifiable monoclonal peak on SPEP with immunofixation electrophoresis (IFE) in 6 patients, and 2 patients had the IgM isotype. No cases of IgD or IgE isotypes were recorded. Only 1 patient’s Ig isotype was not characterized.

**Table 1:** Characteristics of the MM cohort.

Characteristic/parameter	Result		
	*n*	% (*n*/152 ∗ 100)	Mean (SD)
Age (years)			61 (±10)
31–41	4	—	—
42–51	22	—	—
52–61	54	—	—
62–71	50	—	—
72–81	16	—	—
> 81	6	—	—
Sex			
Male	61	40%	—
Female	91	60%	—
M: F	1: 1.5		—
Myeloma‐defining event (MDE)			
Anemia (Hb < 10 g/dL)	102	67%	—
Renal insufficiency (creatinine > 177 μmol/L)	34	22%	—
Hypercalcemia at presentation, before or on the day of biopsy (within 2–4 weeks)	51	34%	—
Hypercalcemia (ca > 2.75) at the time of biopsy	19	13%	—
Clonal BM plasma cells ≥ 60% (determined by IHC or FC)	95	63%	—
SFLC involved‐to‐uninvolved ≥ 100	68	45%	—
Reported BMPC % by each method			
FC PC%	—	—	9.932 (±12.135)
BMA PC%	—	—	30.968 (±21.894)
BMT PC%	—	—	59.054 (±29.155)

The following are the group means and standard deviations for each of FC PC% (mean = 9.932 [SD ± 12.135]), BMA PC% (30.968 [SD ± 21.894]), and BMT PC% (59.054 [SD ± 29.155]). A two‐tailed independent samples *t*‐test revealed a statistically significant difference (*t*[206] = −6.306, *p* < 0.0001, 95% CI: [−23.057, −12.074]) between the group means for FC PC% and BMA PC% (mean difference of −17.566). A two‐tailed independent samples *t*‐test revealed a statistically significant difference (*t*[207] = −8.790, *p* < 0.0001, 95% CI: [−38.785, −24.574]) between the group means for BMA PC% and BMT PC% (mean difference of −31.679).

### 3.2. PLS‐R Model to Predict BMT PC% Using Peripheral Blood Biomarkers

PLS‐R analysis was performed on the training dataset (*n* = 112) to predict BMT PC% using multiple biochemical variables.

**Figure 1:**
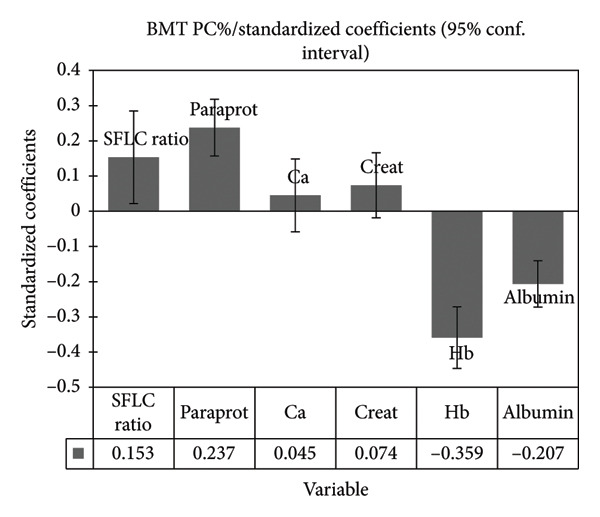
Standardized coefficients for each variable.

The standardized coefficients (SCs) shown in Figure 1 illustrate the influence of BMT PC% on the variables. The BMT PC% had a minor influence on both serum calcium (SC = 0.045) and creatinine levels (SC = 0.074), and the confidence intervals cross zero, which suggests that these effects are not statistically significant. The correlation matrix between BMT PC% and the variables is presented in Table S1.

**Figure 2:**
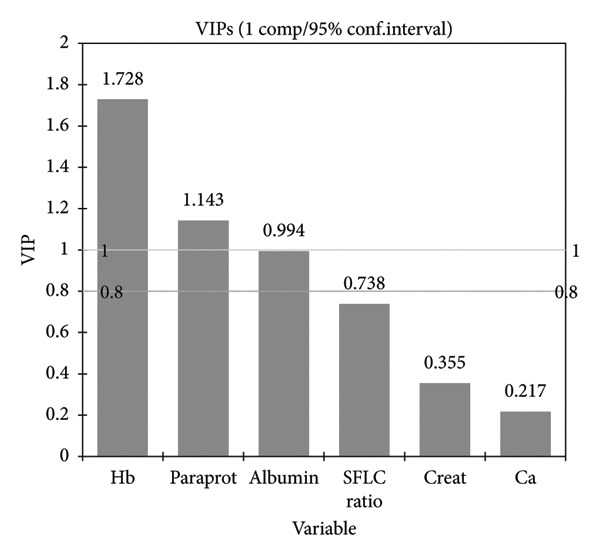
Variable importance in the projection for each variable.

VIP scores of each variable in the projection used in a PLS‐R model are shown in Figure 2. Serum calcium and creatinine had very low VIP scores of 0.217 and 0.355, respectively, statistically insignificant SCs, and were therefore not included in the final prediction model.

The final PLS‐R was then performed on the prioritized explanatory variables (*X*), which included the Hb level measured in g/dL, the measured monoclonal protein on SPEP (paraprotein), serum albumin (albumin), and the involved‐to‐uninvolved SFLC ratio.

### 3.3. Equation of the Model

(1) BMT PC%=113.0499148357416.75950763411998030.2425760505851644.346224636557770.782182472212336+E−∗SFLC ratio+∗paraprotein−∗Hb−∗albumin.

### 3.4. Model Quality

The model quality was tested by using the *Q* ^2^ cumulated index (0.410), *R* ^2^ *Y* cumulated index (0.432), and *R* ^2^ *X* cumulated index (0.470) [21]. The *Q* ^2^ cum represents the cumulative predictive ability of the model, essentially indicating how well the model predicts new data (global goodness of fit) and, in our case, BMT PC% from explanatory variables [22]. A value of 0.410 means that the model explains 41% of the variance in the dependent variable for new data. This value should ideally be close to 1, but typically values above 0.5 indicate a strong predictive capability [21, 22]. An *R* ^2^ *X* value of 0.470 indicates that the model explains 47% of the variance in the predictors (X), suggesting that the selected components moderately capture the X‐space.

### 3.5. BMT PC% Predictions of the Model on the Test Dataset (Validation/Testing the Model)

The Shapiro–Wilk test (*W* = 0.877, *p* < 0.0001), Anderson–Darling test (*A* ^2^ = 5.149, *p* < 0.001), Lilliefors test (*D* = 0.204, *p* < 0.001), and the Jarque–Bera test (JB = 12.243, *p* 0.002), all indicate that the data do not follow a normal distribution.

**Figure 3:**
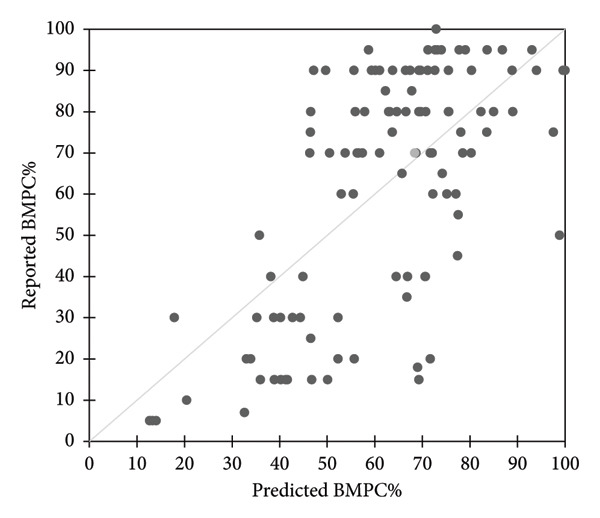
Spearman correlation coefficient to show the relationship between predicted and reported BMT PC% using the PLS‐R equation model.

Spearman correlation coefficient (*ρ* = 0.585, 95% CI: 0.436–0.703, *p* < 0.0001) was calculated to determine the relationship between reported BMT PC% and predicted BMT PC% using the model, which indicated a moderate positive monotonic relationship, a reasonable level of agreement (Figure 3). The two‐tailed test/*t*‐test for two paired samples showed a difference of −0.068 (95% CI: −4.12–3.99, *p* value 0.974, alpha = 0.05).

### 3.6. Regression Analysis to Predict BMT PC% Using Reported FC PC% and BMA PC%

**Figure 4:**
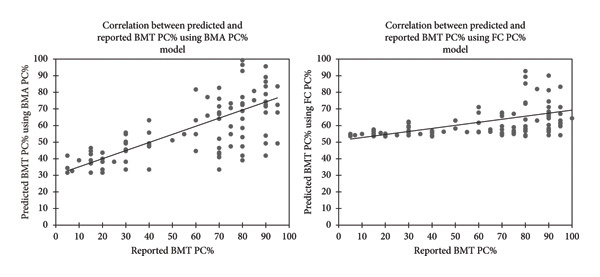
Spearman correlation coefficient to show the relationship between predicted and reported BMT PC% using the linear regression models for reported BMA and FC PC%.

BMA PC% predicts BMT PC% with an *R* ^2^ of 0.489 (moderate accuracy) with the linear regression equation model, while FC PC% offers a much lower predictive power (*R* ^2^) of 0.184. Analysis of variance (ANOVA) showed that the predictor variable, BMA PC%, significantly contributes to explaining variations in BMT PC% with an F‐statistic of 87.899 and a *p* value of < 0.0001. The linear regression model using BMA PC% has a mean absolute percentage error (MAPE) of 65.457, which indicates considerable variability. The Spearman correlation coefficient (*p* = 0.721, 95% CI: 0.591–0.815) of the predicted BMT and reported BMT PC%, using the BMA PC%, is shown in Figure 4. The Spearman correlation coefficient when using FC PC% to predict BMT PC% (*p* = 0.541, 95% CI: 0.386–0.667) is also shown in Figure 4. The equations as well as a summary of the quality of the prediction models are presented in Table S2.

## 4. Discussion

### 4.1. PLS‐R Analysis to Predict BMT PC% Using Peripheral Blood Biomarker Variables

All explanatory variables included in the final prediction model show statistically significant contributions to the prediction model, as none of the confidence intervals of the SCs pass through zero. BMT PC% appears to have the most substantial influence on Hb levels, as the SC is highly negative, meaning that higher BMT PC% is associated with lower Hb. Among the explanatory variables included in the final PLS‐R prediction model, both the ratio of involved‐to‐uninvolved SFLC and the paraprotein level have a positive relationship, while serum albumin and Hb have a negative relationship with the reported BMT PC%.

The cumulative *Q* ^2^ value (*Q* ^2^ cum = 0.410) of this model indicates a moderate predictive performance, suggesting that although the current model has acceptable explanatory (*R* ^2^ *Y* = 0.432) and predictive capabilities, there is still much room for improvement. The relatively small gap between *R* ^2^ *Y* and *Q* ^2^ cum reflects good internal consistency between model fit and prediction. Taken together, these results support the model as a valid proof of concept for estimating the BMT PC% using a multivariate surrogate approach. With methodological refinements, such as the inclusion of additional latent components, improved strategies for data preprocessing, or alternative modeling techniques, the predictive power of the model could possibly be enhanced. The *R* ^2^ *X* value (0.470) also suggests that almost half of the variance in the explanatory variables is accounted for, with the remaining variance likely to be due to unmeasured or complex clinical factors, including comorbidities (e.g., systemic hypertension) and therapeutic interventions.

The laboratory values used in the model may not only reflect the extent of the disease but may also be influenced by the patient’s treatment strategies such as intravenous fluid administration or blood transfusions. Hb was the only parameter routinely measured at the time of BM biopsy, whereas biochemical markers were collected within a 2‐week window before or after the biopsy. Serum calcium, in particular, is difficult to use in predictive models, as emergency treatment of hypercalcemia takes precedence over the timing of the BM biopsy. As a result, serum calcium values at the time of biopsy may not accurately reflect disease severity. This is evident in our study population, in which only 13% of patients had myeloma‐defining hypercalcemia at the time of the BM biopsy, compared to 34%, when values obtained within 2 weeks before or after the biopsy were considered. Importantly, serum calcium and creatinine were not included in the final model due to their nonspecificity and susceptibility to treatment, or comorbidity‐induced variation. This emphasizes the need to prioritize disease‐specific and stable biomarkers, while careful adjustment for comorbidities may help to mitigate these limitations and improve accuracy in future models.

### 4.2. BMT PC% Predictions Using the PLS‐R Model

The observed moderately positive monotonic relationship between reported and predicted BMPC% supports the potential utility of the PLS‐R model in approximating tumor burden in the BM. The close agreement between the two values, reinforced by the lack of a statistically significant difference in pairwise comparisons, emphasizes the consistency and practical relevance of the model. Although the agreement is not perfect, this level of correlation is encouraging for a preliminary model and suggests a solid basis on which to develop more refined predictive tools.

### 4.3. Regression Analysis to Predict BMT PC% Using FC PC% and BMA PC%

BMA PC% showed moderate accuracy in predicting the BMT PC% and performed significantly better than estimates obtained from FC PC%. The strong statistical significance of BMA PC% as a predictor of BMT PC% and the higher correlation coefficient emphasize the potential value of BMA as a practical surrogate, particularly when trephine biopsies are suboptimal or unavailable, which is not uncommon in patients with advanced MM. However, the relatively high MAPE highlights the considerable variability at the individual level, suggesting that while the BMA PC% may be useful for an approximate assessment of BMT PC%, it should not be relied upon exclusively for accurate quantification. Nonetheless, it can serve as a useful screening tool to identify cases that are likely to exceed clinically significant thresholds (e.g. ≥ 60% PC infiltration) relevant for diagnostic and prognostic stratification.

### 4.4. Limitations and Future Considerations

This analysis was mainly performed on diagnostic BM biopsies to allow the evaluation of the corresponding FC values, which in our center is mainly performed only in the diagnosis of PCN. It therefore remains unclear whether the results are directly transferable to follow‐up BM samples, as this model has not been tested in this way. This model is also not applicable to patients with nonsecretory myeloma, who represent a small but important minority (∼3% of cases), as there are no paraprotein‐based parameters in this setting.

One of the limitations is the lack of documentation as to whether FC was performed on samples from the first or second draw during BMA collection. It is well known that subsequent collections or aspirates of more than 1 mL can cause hemodilution [23–25]. However, consistent with previous reports, the percentage of PCs reported by FC (FC PC%) in our study was consistently lower than in myelograms performed on BMAs (BMA PC%) or BMT PC%, and the BMA PC% was consistently lower than BMT PC% [14, 15, 25–27].

Another limitation is the potential for greater variation in predictive modeling between laboratories and institutions due to differences in biochemical assays, FC protocols, and reporting practices. While our single‐center design ensured methodological consistency, this limits generalizability, and external validation between centers with harmonized protocols will be essential for wider clinical application.

Several factors should be addressed to improve future predictive models.

1. Standardization of sample collection: The variability in sample collection, including peripheral blood collection for biomarker testing, aspirate volume, and choice of collection for FC, must be minimized. The initial collection (first draw aspirate) should be standardized for FC testing to ensure accuracy of FC PC% measurements.
2. Synchronize biochemical tests: BM biopsy should be performed on the same day as biochemical tests to improve correlation and reliability of the prediction models.
3. Standardize PC counting: The BMT PC% should be uniformly determined using a published, validated manual counting method, e.g., by Gantana et al. [17].
4. Clinical data collection: Comprehensive documentation of comorbidities, such as hypertension, hyperparathyroidism, diabetes, and inflammation, that influence the biochemical values used in this model should be included to allow for appropriate adjustments in the statistical analysis.
5. Prospective data collection would be more effective than the retrospective methods used in this study. In addition, incorporating other MDEs, such as osteolytic lesions, could further enhance the model’s accuracy.

Despite these limitations, this study provides a valuable proof of concept for the use of biomarker values, FC, and the percentage of PCs from the BMA myelogram to predict BMT PC% and thus offers potential for future disease monitoring in PCNs. Such a prediction model could help minimize patient pain and discomfort during BM biopsies while also reducing medical care costs. The results of this study also provide meaningful insights into hematological assessment and the relationships between PC quantification methods. Until such prediction models are refined, evaluation of BM biopsies by an experienced hematopathologist will remain the gold standard for determining and monitoring BMT PC% in PCNs at diagnosis and follow‐up.

## 5. Conclusion

While these current predictive models are of moderate quality, this study shows that the development of a robust and reliable model is feasible with standardized data collection and an improved methodology. Key factors include the consistent determination of the percentage of PCs in the BMAs and BMTs and the timing of the determination of biomarkers. Prospective studies designed with these considerations in mind will significantly increase predictive accuracy and clinical utility. Achieving this goal will require close collaboration between clinicians and pathologists in multiple centers to ensure harmonized sampling, biomarker measurement protocols, and comprehensive integration of clinical data.

### 5.1. Key Message

Despite its limitations and the moderate predictive quality of the models, this study represents a valuable proof of concept and highlights the potential of predictive models to complement hematopathologists’ assessments in disease surveillance of PCNs.

## Ethics Statement

Ethical approval was granted by the Health Research Ethics Committee of Stellenbosch University (HREC reference number: N24/09/107). Individual patient consent was waived as this study poses no potential risk to patients and samples were de‐identified.

## Consent

Please see the Ethics Statement.

## Disclosure

Preliminary findings from this work were previously presented in an abstract form at the 2025 International Society for Laboratory Hematology (ISLH) Annual Conference as poster P190 by Ethan James Gantana and Zivanai Cuthbert Chapanduka. A Novel Strategy for Assessing Bone Marrow Plasma Cell Percentage: Development and Internal Validation of a Surrogate Calculation Approach available at: https://www.researchgate.net/publication/391590557_P190_ISLH_2025.

## Conflicts of Interest

The authors declare no conflicts of interest.

## Author Contributions

Ethan James Gantana conceptualized and designed the study, conducted the data analysis, created the graphic presentations, and coordinated all aspects of the implementation. Ethan James Gantana also wrote the original manuscript and participated in additional reviews. Zivanai Cuthbert Chapanduka wrote the second draft and conducted the final review of the manuscript.

## Funding

No funding was received for this study.

## Supporting Information

Additional supporting information can be found online in the Supporting Information section.

## Supporting information


**Supporting Information 1** Figure S1: Flow diagram of the study population.

**Supporting Information 2** Table S1: Correlation matrix for all variables.

**Supporting Information 3** Table S2: Equations and summary of the quality of the prediction models.

## Data Availability

The data that support the findings of this study are available from the corresponding author upon reasonable request.

## References

[1] Swerdlow S. H. , Campo E. , Pileri S. A. et al., WHO Classification of Tumours of the Hematopoietic and Lymphoid Tissues the 2016 Revision of the World Health Organization Classification of Lymphoid Neoplasms, Blood. (2016) 127. 10.1182/blood-2016-01-643569 PMC4874220 26980727

[2] Kumar S. , Paiva B. , Anderson K. C. et al., International Myeloma Working Group Consensus Criteria for Response and Minimal Residual Disease Assessment in Multiple Myeloma, The Lancet Oncology. (2016) 17, no. 8, e328–e346, 10.1016/S1470-2045(16)30206-6, 2-s2.0-84995917601. 27511158

[3] Rajkumar S. V. , Fonseca R. , Dispenzieri A. et al., Methods for Estimation of Bone Marrow Plasma Cell Involvement in Myeloma: Predictive Value for Response and Survival in Patients Undean rgoing Autologous Stem Cell Transplantation, American Journal of Hematology. (2001) 68, no. 4, 269–275, 10.1002/ajh.10003, 2-s2.0-0035196437. 11754416

[4] Child J. A. , Anderson K. , Barlogie B. et al., Criteria for the Classification of Monoclonal Gammopathies, Multiple Myeloma and Related Disorders: A Report of the International Myeloma Working Group, British Journal of Hematology. (2003) 121, no. 5, 749–757, 10.1046/j.1365-2141.2003.04355.x, 2-s2.0-0038509089. 12780789

[5] Hutchison C. A. , Batuman V. , Behrens J. et al., The Pathogenesis and Diagnosis of Acute Kidney Injury in Multiple Myeloma, Nature Reviews Nephrology. (2012) 8, no. 1, 43–51, 10.1038/nrneph.2011.168, 2-s2.0-84455205655. PMC3375610 22045243

[6] Rajkumar S. V. , Dimopoulos M. A. , Palumbo A. et al., International Myeloma Working Group Updated Criteria for the Diagnosis of Multiple Myeloma, The Lancet Oncology. (2014) 15, no. 12, e538–e548, 10.1016/S1470-2045(14)70442-5, 2-s2.0-84908604358. 25439696

[7] Larsen J. T. , Kumar S. K. , Dispenzieri A. , Kyle R. A. , Katzmann J. A. , and Rajkumar S. V. , Serum Free Light Chain Ratio as a Biomarker for High-Risk Smoldering Multiple Myeloma, Leukemia. (2013) 27, no. 4, 941–946, 10.1038/leu.2012.296, 2-s2.0-84876141787. 23183428 PMC3629951

[8] Dispenzieri A. , Kyle R. A. , Katzmann J. A. et al., Immunoglobulin Free Light Chain Ratio is an Independent Risk Factor for Progression of Smoldering (Asymptomatic) Multiple Myeloma, Blood. (2008) 111, no. 2, 785–789, 10.1182/blood-2007-08-108357, 2-s2.0-38349136782. 17942755 PMC2200851

[9] Rajkumar S. V. , Kyle R. A. , Therneau T. M. et al., Serum Free Light Chain Ratio is an Independent Risk Factor for Progression in Monoclonal Gammopathy of Undetermined Significance, Blood. (2005) 106, no. 3, 812–817, 10.1182/blood-2005-03-1038, 2-s2.0-23044481861. 15855274 PMC1895159

[10] Rajkumar S. V. , Multiple Myeloma: 2022 Update on Diagnosis, Risk Stratification, and Management, American Journal of Hematology. (2022) 97, no. 8, 1086–1107, 10.1002/ajh.26590. 35560063 PMC9387011

[11] Lidén Y. , Landgren O. , Arnér S. , Sjölund K. , and Johansson E. , Procedure-Related Pain Among Adult Patients With Hematologic Malignancies, Acta Anaesthesiologica Scandinavica. (2009) 53, no. 3, 354–363, 10.1111/j.1399-6576.2008.01874.x, 2-s2.0-60349088690. 19243321 PMC6889807

[12] Degen C. , Christen S. , Rovo A. , and Gratwohl A. , Bone Marrow Examination: A Prospective Survey on Factors Associated With Pain, Annals of Hematology. (2010) 89, no. 6, 619–624, 10.1007/s00277-010-0934-0, 2-s2.0-77951642463. 20333524

[13] Smock K. J. , Perkins S. L. , and Bahler D. W. , Quantitation of Plasma Cells in Bone Marrow Aspirates by Flow Cytometric Analysis Compared with Morphologic Assessment, Archives of Pathology & Laboratory Medicine. (2007) 131, no. 6, 951–955, 10.5858/2007-131-951-qopcib. 17550325

[14] Al-Quran S. Z. , Yang L. , Magill J. M. , Braylan R. C. , and Douglas-Nikitin V. K. , Assessment of Bone Marrow Plasma Cell Infiltrates in Multiple Myeloma: The Added Value of CD138 Immunohistochemistry, Human Pathology. (2007) 38, no. 12, 1779–1787, 10.1016/j.humpath.2007.04.010, 2-s2.0-36549041418. 17714757 PMC3419754

[15] Smith F. B. and Elnawawi A. , Technical Note: A Counting Strategy for Estimating Plasma Cell Number in CD138-Stained Bone Marrow Core Biopsy Sections, Annals of Clinical Laboratory Science. (2008) 38, no. 2. 18469359

[16] Gebreslassie K. S. , Bassa F. C. , Chapanduka Z. C. , and Warwick J. M. , The Relationship Between Bone Marrow Involvement on 18F-FDG PET/CT and Bone Marrow Biopsy in Patients with Multiple Myeloma and Other Plasma Cell Neoplasms, South African Journal of Oncology. (2022) 6, 10.4102/sajo.v6i0.197.

[17] Gantana E. , Mashigo N. , Abdullah I. et al., Evaluation of an Innovative New Method for Quantitation of Plasma Cells on CD138 Immunohistochemistry, Journal of Clinical Pathology. (2023) 76, no. 4, 261–265, 10.1136/jclinpath-2021-207828. 34625512

[18] Gantana E. J. , Nell E. , Musekwa E. et al., Evaluation of a New Technique Using Artificial Intelligence for Quantification of Plasma Cells on CD138 Immunohistochemistry, The International Journal of Literary Humanities. (2023) 46, no. 1, 50–57, 10.1111/ijlh.14161. 37621174

[19] Gantana E. J. , Musekwa E. , and Chapanduka Z. C. , Advances in Estimating Plasma Cells in Bone Marrow: A Comprehensive Method Review, African Journal of Laboratory Medicine. (2024) 13, no. 1, 10.4102/ajlm.v13i1.2381. PMC11304106 39114749

[20] Addinsoft , {XLSTAT} Statistical and Data Analysis Solution. {Paris}, {France}. XLSTAT, Your data analysis solution, 2021.

[21] Geladi P. and Kowalski B. R. , Partial Least-Squares Regression: A Tutorial, Analytica Chimica Acta. (1986) 185, no. C, 1–17, 10.1016/0003-2670(86)80028-9, 2-s2.0-11144325691.

[22] Mevik B. H. and Wehrens R. , The Pls Package: Principal Component and Partial Least Squares Regression in R, Journal of Statistical Software. (2007) 18, no. 2, 10.18637/jss.v018.i02, 2-s2.0-33846829987.

[23] Batinić D. , Marusić M. , Pavletić Z. et al., Relationship Between Differing Volumes of Bone Marrow Aspirates and Their Cellular Composition, Bone Marrow Transplantation. (1990) 6, no. 2, 103–107. 2207448

[24] Bain B. J. , Bates I. , Laffan M. A. , and Lewis S. M. , Dacie and Lewis Practical Hematology, 2016.

[25] Jain G. , Das N. , Gajendra S. et al., Effect of the Sequence of Pull of Bone Marrow Aspirates on Plasma Cell Quantification in Plasma Cell Proliferative Disorders, The International Journal of Literary Humanities. (2022) 44, no. 5, 837–845, 10.1111/ijlh.13887. 36106595

[26] Ajise O. E. , Roshal M. , Wang L. et al., Clinical Utility of Morphology, Immunohistochemistry, Flow Cytometry, and FISH Analysis in Monitoring of Plasma Cell Neoplasms in the Bone Marrow, Journal of Hematopathology. (2016) 9, no. 1, 9–18, 10.1007/s12308-015-0264-1, 2-s2.0-84975764210.

[27] Paiva B. , Vidriales M. B. , Perez J. J. et al., Multiparameter Flow Cytometry Quantification of Bone Marrow Plasma Cells at Diagnosis Provides More Prognostic Information Than Morphological Assessment in Myeloma Patients, Hematologica. (2009) 94, no. 11, 1599–1602, 10.3324/hematol.2009.009100. PMC2770972 19880781

