# Supplementary material for: A Novel Strategy for Assessing Bone Marrow Plasma Cell Percentage: Development and Internal Validation of a Surrogate Calculation Approach
Source: Adv Hematol. 2025 Nov 14;2025:1191575. doi: 10.1155/ah/1191575 (PMC12616256; doi:10.1155/ah/1191575)
Supplement: Supplementary file 2 — Supporting Information 2 Table S1: Correlation matrix for all variables. [file AH-2025-1191575-s003.docx]

| **Variables** | SFLC ratio | Paraprotein | Calcium | Creatinine | Hb | Albumin | **BMT PC%** |
| --- | --- | --- | --- | --- | --- | --- | --- |
| SFLC ratio | **1** | -0.241 | 0.106 | 0.136 | -0.121 | 0.149 | **0.267** |
| Paraprotein | -0.241 | **1** | -0.192 | -0.297 | -0.431 | -0.580 | **0.413** |
| Calcium | 0.106 | -0.192 | **1** | 0.111 | 0.037 | 0.252 | **0.079** |
| Creatinine | 0.136 | -0.297 | 0.111 | **1** | -0.249 | -0.052 | **0.128** |
| Hb | -0.121 | -0.431 | 0.037 | -0.249 | **1** | 0.509 | **-0.625** |
| Albumin | 0.149 | -0.580 | 0.252 | -0.052 | 0.509 | **1** | **-0.359** |
| **BMT PC%** | **0.267** | **0.413** | **0.079** | **0.128** | **-0.625** | **-0.359** | **1** |

Table S1: Correlation matrix for all the variables.
