# Supplementary material for: A Novel Strategy for Assessing Bone Marrow Plasma Cell Percentage: Development and Internal Validation of a Surrogate Calculation Approach
Source: Adv Hematol. 2025 Nov 14;2025:1191575. doi: 10.1155/ah/1191575 (PMC12616256; doi:10.1155/ah/1191575)
Supplement: Supplementary file 3 — Supporting Information 3 Table S2: Equations and summary of the quality of the prediction models. [file AH-2025-1191575-s002.docx]

| **Predictor** | **Correlation with BMT PC%** | **Regression Equation** | **R^2^ Value** | **F-test (ANOVA)** | **P-value** |
| --- | --- | --- | --- | --- | --- |
| **FC PC%** | 0.428 | BMT PC%= 53,4380981574473+0,677066040216679*FCPC% | 0.184 | **25.187** | **<0,0001** |
| **BMA PC%** | 0.699 | BMT PC% = 30,6478947258939+0,927394637068824*BMAPC% | 0.489 | 87.899 | **<0,0001** |

Table S2: Equations and summary of the quality of the prediction models.
