# Supplementary material for: A Novel Strategy for Assessing Bone Marrow Plasma Cell Percentage: Development and Internal Validation of a Surrogate Calculation Approach
Source: Adv Hematol. 2025 Nov 14;2025:1191575. doi: 10.1155/ah/1191575 (PMC12616256; doi:10.1155/ah/1191575)
Supplement: Supplementary file 3 [file AH-2025-1191575-s001.docx]

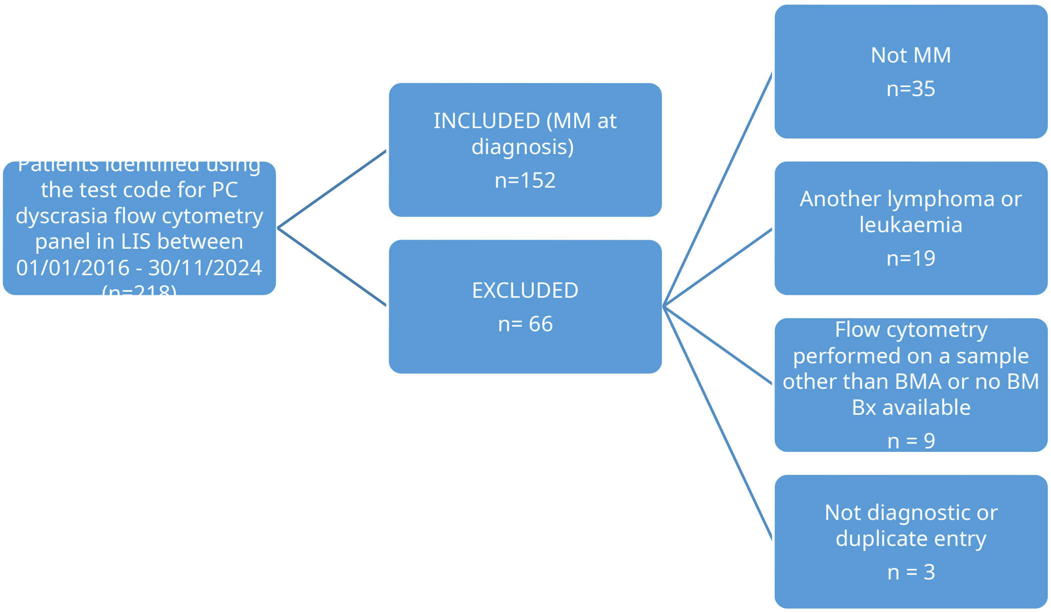
Patients identified using the test code for PC dyscrasia flow cytometry panel in LIS between 01/01/2016 - 30/11/2024 (n=218)
INCLUDED (MM at diagnosis) n=152
EXCLUDED n= 66
Not MM n=35
Another lymphoma or leukaemia n=19
Flow cytometry performed on a sample other than BMA or no BM Bx available n = 9
Not diagnostic or duplicate entry n = 3
